# The prevalence of cardiovascular disease in adults with type 2 diabetes mellitus in Saudi Arabia - CAPTURE study

**DOI:** 10.15537/smj.2023.44.1.20220402

**Published:** 2023-01

**Authors:** Abdullah M. Alguwaihes, Amani Alhozali, Moataz M. Yahia, Tarek Abdel-Nabi, Mohamed Hassan Hatahet, Nader I. Albalkhi, Saud Al Sifri

**Affiliations:** *From the Department of Internal Medicine (Alguwaihes), King Saud University, from the Department of Internal Medicine (Alguwaihes), King Saud University Medical City, from the Department of Medical Affairs (Yahia, Abdel-Nabi), Novo Nordisk, from the Department of Internal Medicine (Albalkhi), Specialized Medical Centre Hospital, Riyadh, from the Department of Internal Medicine (Alhozali), King Abdulaziz University Hospital, Jeddah, from the Department of Internal Medicine (Hatahet), King Abdulaziz Hospital for National Guard, Al Ahsa, and from the Department of Internal Medicine (Al Sifri), Al Hada Military Hospital, Taif, Kingdom of Saudi Arabia.*

**Keywords:** cardiovascular system, epidemiology, type 2 diabetes mellitus, Saudi Arabia

## Abstract

**Objectives:**

To investigate cardiovascular disease (CVD) prevalence in adult patients with type 2 diabetes mellitus (T2DM) in Saudi Arabia using data from the CAPTURE cross-sectional study.

**Methods:**

CAPTURE was a non-interventional, multinational study carried out between December 2018 and September 2019. In Saudi Arabia, clinical (including medication) and demographic data were collected across 7 sites (Alhada Armed Forces Hospital, Taif; King Saud University Medical City, King Saud University, Riyadh; Specialized Medical Centre Hospital, Riyadh; King Abdulaziz University Hospital, Jeddah; King Abdulaziz Hospital for National Guard, Al Ahsa; Diabetes and Endocrinology Center, Buraidah; and Dallah Hospital, Riyadh, Saudi Arabia) from adults aged ≥18 years. The prevalence of CVD was estimated and weighted according to care setting, with data between groups not statistically compared.

**Results:**

Among the 883 adults enrolled in this study (566 from primary care, 317 from secondary care), 158 had established CVD, making the weighted prevalence of 18% (95% CI: [15.5-20.5]). The weighted prevalence of atherosclerotic CVD was 15.1% (95% CI: [12.8-17.5]), accounting for 82.4% of the CVD cases. Coronary heart disease was the most common subtype of CVD (13.4%), followed by cerebrovascular disease (1.7%). A total of 23.6% of patients were treated with glucose-lowering agents with proven cardiovascular benefit.

**Conclusion:**

In Saudi Arabia, approximately one in 5 adults with T2DM had established CVD, lower than the global prevalence, possibly because of disparities in patient characteristics, potential genetic predispositions, or a lack of accurate documentation due to poor coordination between care settings.

**T**ype 2 diabetes mellitus (T2DM) is becoming increasingly prevalent around the globe; between 2000 and 2019 the prevalence of diabetes tripled, rising from approximately 151 million (4.6% of the global population at the time) to 463 million (9.3%) cases in adults aged 20-79 years, of which T2DM accounted for 90%.^1^ The World Health Organization (WHO) estimated that in 2016, 14.4% of adults aged 18 years and older in the Saudi Arabian population had diabetes, which had risen to 18.3%, of adults aged 20-79 years, in 2019.^1,2^

Type 2 diabetes mellitus is known to cause several micro- and macrovascular complications, such as cardiovascular disease (CVD).^1^ Literature throughout the last decade has suggested that the CVD risk factor prevalence in Saudi Arabia is high.^3-6^ The recent Prospective Urban Rural Epidemiology (PURE) study in Saudi Arabia found the prevalence of obesity to be as high as 49.6%, 32.1% in dyslipidemia, 30.3% in hypertension, and 25.2% in diabetes.^7^ These findings can be partially explained by the recent rapid economic growth of Saudi Arabia and its neighboring Gulf countries; increasingly people are eating poorer quality food and engaging in less physical exercise than previous generations.^8^ A survey from 2018 revealed that 82.6% of people surveyed in Saudi Arabia were physically inactive.^9^ In addition, 88% of people in Saudi Arabia have reported that they consume less than the recommended 5 portions of fruit and vegetables each day.^4^ In the same study, over two-thirds of the study population had obesity or were overweight.^4^

Due to the high CVD risk factor prevalence and rising prevalence of T2DM in Saudi Arabia, it is believed that CVD is also becoming increasingly prevalent.^7^ The potential rise in the number of patients with CVD is of concern due to its association with an additional risk of disability, morbidity, and mortality.^10^ In addition, CVD represents the leading cause of death (37%) and is the leading cause of disability-adjusted life-years in Saudi Arabia.^11,12^ Specifically, data from 2015 showed that the national burden of CVD in Saudi Arabia was in the range of 6601-7500 cases per 100,000.^10^ However, interestingly, the recent PURE study from Saudi Arabia found the prevalence of some CVD subtypes to be low, such as ischemic heart disease (2.5%), stroke (1%), and heart failure (0.6%).^7^ Due to the currently lacking and conflicting data available, there is a need to further explore the prevalence of CVD in patients with T2DM in Saudi Arabia.

As it is now well established that T2DM is a contributor to CVD, treatment guidelines from international organizations have been adapted to better reflect this.^13^ It is recommended that following treatment with metformin, patients with T2DM and concurrent CVD or at high risk of developing CVD should be treated with glucose-lowering agents (GLAs) with proven cardiovascular (CV) benefit, such as glucagon-like peptide-1 receptor agonists (GLP-1 RAs) and sodium-glucose co-transporter-2 inhibitors (SGLT2is).^13^ The CV benefit of these medications has been shown in previous CV outcome trials (for example, the LEADER trial for liraglutide, SUSTAIN 6 trial for semaglutide, REWIND trial for dulaglutide, EMPA-REG OUTCOME trial for empagliflozin, DECLARE-TIMI 58 trial for dapagliflozin, and CANVAS trial for canagliflozin).^14-19^ Aligned with these CV outcome trial results, the Saudi Society of Endocrinology and Metabolism (SSEM) published a consensus statement, which included the recommendation of using GLP-1 RAs and SGLT2is in patients who have inadequately controlled glycated hemoglobin (HbA1c) following metformin therapy and have a history of atherosclerotic CVD (ASCVD) and heart failure.^20^ Measuring the prevalence of CVD in patients with T2DM will enable an assessment of these guidelines in the coming years.

The primary objective of this cross-sectional study was to investigate the prevalence of CVD among patients with T2DM across both primary and secondary care in Saudi Arabian participants of the multinational CAPTURE study.^21^ We also investigated the use of GLAs with proven CV benefit, including GLP-1 RAs (dulaglutide, liraglutide, and semaglutide) and SGLT2is (canagliflozin, dapagliflozin, and empagliflozin).

## Methods

CAPTURE, a non-interventional and cross-sectional study, was carried out at 214 centers across 13 countries, from December 2018 until September 2019. The study design has been reported previously in the primary CAPTURE study, where the global findings have been detailed.^21^ The focus of this report is the findings from Saudi Arabia.

The study was carried out in accordance with the Declaration of Helsinki and the International Society for Pharmacoepidemiology Good Pharmacoepidemiology Practices.^22,23^ Informed consent was obtained from all patients prior to taking part in the study; the clinical research ethics committees approved the study protocol and informed consent form (Appendix 1).

Study site selection was advised by local medical affairs specialists who, based on desk research and years of liaising with healthcare professionals, provided information on the types of care available in Saudi Arabia (such as primary and secondary care), as well as on the different kinds of professionals that provide diabetes care (such as primary care practitioners, endocrinologists, diabetologists, and cardiologists). Geographical spread of sites and the division of patients being treated at private (namely, Specialized Medical Centers and Dallah Hospitals, Riyadh) and public centers (namely, all other sites) were also considered. Based on this information, the contract research organization selected the final participating sites, which were approved by the sponsor. Sites were selected to be as representative as possible of the country as a whole to try to limit selection bias. The sites chosen were: Alhada Armed Forces Hospital, Al Hada; King Saud University Medical City, King Saud University, Riyadh; Specialized Medical Centre Hospital, Riyadh; King Abdulaziz University Hospital, Jeddah; King Abdulaziz Hospital for National Guard, Al Ahsa; Diabetes and Endocrinology Center-Buraidah, Buraidah; Dallah Hospital, Riyadh.^21^

As outlined in the primary publication,^21^ patients 18 years of age or older who had a diagnosis of T2DM ≥180 days prior to the start of the study were enrolled over a 90-day period. Exclusion criteria included diagnosis of type 1 diabetes or congenital heart disease or malformation, and mental incapacity or language barriers that prevented an appropriate understanding of what the study entailed.

Data were collected by physicians or appropriately qualified delegates using standardized electronic case report forms, which were then transferred to a central database. Relevant data were extracted from patients’ medical records. The data collected included information regarding the patients’ overall health status (such as medical history and clinical parameters), demography and use of CV medication or GLAs. If there was no information relating to certain medical histories in their records (such as neuropathy, nephropathy, or retinopathy), patients were asked if they had been previously diagnosed with them.

Patients were categorized as having established CVD if their medical records indicated the diagnosis of any of the following conditions: aortic disease, cardiac arrhythmia or conduction abnormalities, carotid artery disease, cerebrovascular disease, coronary heart disease, heart failure, or peripheral artery disease. Patients were categorized as having ASCVD if they had any of the listed conditions: cerebrovascular disease, coronary heart disease, peripheral artery disease, or carotid artery disease. Full definitions for each of the above CVD subtypes are shown in Appendix 2.

To calculate the sample size, the prevalence of those with T2DM and CVD was assumed to be 25-40% and a sample size of 800 was selected to result in a precision of ±2-3% points of the calculated CVD prevalence.

### Statistical analysis

The prevalence of CVD and the aforementioned subtypes in patients with T2DM was calculated, with 95% confidence interval (CI). The estimates for Saudi Arabia were weighted according to care setting (using primary and secondary settings). The data are descriptive and the differences between groups were not compared statistically.

SAS, version 9.4 (SAS Institute, Cary, NC, USA) was used to undertake these statistical analyses.

## Results

Between December 2018 and September 2019, 883 patients from 7 sites were enrolled in the study, of which 566 were treated in primary care and 317 were treated in secondary care. The median age (interquartile range) of the study population was 58 years (50-65), the median body mass index (BMI) was 29.1 kg/m^2^ (26.5-32.9), 8.1% of patients had an estimated glomerular filtration rate (eGFR) of <59 mL/minute/1.73 m^2^ and 80.9% were ‘never smokers’ (Table 1).

Some patient characteristics were markedly different between the primary and secondary care groups (Table 1). Patients in primary care had slightly longer median diabetes duration (11.7 versus 8.3 years), an increased median BMI (30.2 versus 27.9 kg/m^2^) and fewer were female (38.7% versus 58.4%) than in secondary care. Similarly, 10.5% of patients in primary care had an eGFR of <59 mL/min/1.73 m^2^ versus 4.4% in secondary care. Conversely, a larger proportion in primary care were ‘never smokers’ (84.5% versus 74.4%), and fewer had microalbuminuria (19.6% versus 33%) than in secondary care. More patients had a medical history of hypertension (68.2% versus 32.9%) and microvascular complications, such as retinopathy (18.7% versus 5.4%), nephropathy (15% versus 1.9%), and neuropathy (15.5% versus 5.7%) in the primary versus secondary care group.

**Figure 1:**
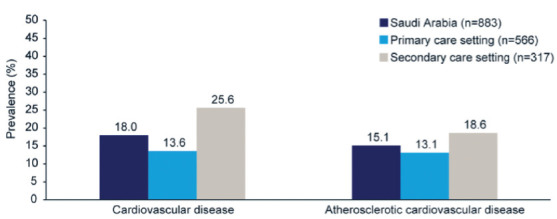
- Cardiovascular disease and atherosclerotic cardiovascular disease prevalence in Saudi Arabia, stratified by care setting. Differences between care settings were not compared statistically.

Of the 883 patients enrolled, 158 had established CVD, making the weighted prevalence 18% (95% CI: [15.5-20.5]; Figure 1). The weighted prevalence of ASCVD was 15.1% (95% CI: [12.8,17.5]); ASCVD accounted for 84.2% of the CVD cases. Coronary heart disease was the most common CVD subtype (13.4%), followed by cerebrovascular disease (1.7%), and heart failure (0.9%). The prevalence of each of the CVD subtypes is illustrated in Appendix 3.

**Table 1:** - Characteristics of patients in the CAPTURE Saudi Arabia by care setting.

Characteristics	Study population (N=883)		Care setting			
			Primary care (n=566)		Secondary care (n=317)	
	n	Data	n	Data	n	Data
Female	883	404 (45.8)	566	219 (38.7)	317	185 (58.4)
Age, years	883	58 [50-65]	566	60 [53-66]	317	54 [48-62]
Diabetes duration, years	880	10.0 [5.0-17.0]	563	11.7 [5.1-20.0]	317	8.3 [4.5-12.8]
HbA_1c_, %	876	8.0 [7.1-9.0]	559	7.8 [6.8-9.1]	317	8.4 [7.8-9.0]
HbA_1c_, mmol/mol	876	64 [54-75]	559	62 [51-76]	317	68 [62-75]
BMI, kg/m^2^	881	29.1 [26.5-32.9]	564	30.2 [26.5-34.3]	317	27.9 [26.5-30.0]
Systolic blood pressure, mmHg	882	132 [122-143]	565	134 [123-145]	317	130 [122-140]
Diastolic blood pressure, mmHg	882	76 [69-80]	565	75 [69-80]	317	76 [69-82]
Hypertension	876	486 (55.5)	560	382 (68.2)	316	104 (32.9)
Familial hypercholesterolemia	552	112 (20.3)	294	90 (30.6)	258	22 (8.5)
***eGFR, mL/min/1.73 m*** ^2^	814		497		317	
>89		318 (39.1)		222 (44.7)		96 (30.3)
>59-89		430 (52.8)		223 (44.9)		207 (65.3)
>29-59		60 (7.4)		48 (9.7)		12 (3.8)
≤29		6 (0.7)		4 (0.8)		2 (0.6)
***Albuminuria***	668		474		194	
Normal to mildly increased		470 (70.4)		340 (71.7)		130 (67.0)
Microalbuminuria		157 (23.5)		93 (19.6)		64 (33.0)
Macroalbuminuria		41 (6.1)		41 (8.6)		-
***Retinopathy***	883		566		317	
Yes		123 (13.9)		106 (18.7)		17 (5.4)
Yes (referred by patient)		11 (1.2)		10 (1.8)		1 (0.3)
No		749 (84.8)		450 (79.5)		299 (94.3)
***Nephropathy***	883		566		317	
Yes		91 (10.3)		85 (15.0)		6 (1.9)
Yes (referred by patient)		24 (2.7)		23 (4.1)		1 (0.3)
No		768 (87.0)		458 (80.9)		310 (97.8)
***Neuropathy***	883		566		317	
Yes		106 (12.0)		88 (15.5)		18 (5.7)
Yes (referred by patient)		24 (2.7)		24 (4.2)		-
No		753 (85.3)		454 (80.2)		299 (94.3)
***Smoking***	883		566		317	
Current smoker		78 (8.8)		63 (11.1)		15 (4.7)
Previous smoker		91 (10.3)		25 (4.4)		66 (20.8)
Never smoker		714 (80.9)		478 (84.5)		236 (74.4)
Physical activity, days/week*	486		284		202	
0-1		231 (47.5)		185 (65.1)		46 (22.8)
2-3		144 (29.6)		48 (16.9)		96 (47.5)
4-5		76 (15.6)		20 (7.0)		56 (27.7)
6-7		35 (7.2)		31 (10.9)		4 (2.0)

Values are presented as numbers and precentages (%) or median interquartile range [IQR]. Differences between the care settings of primary and secondary were not compared statistically.

^*^ Number of days in the last week with a minimum of 30 minutes of moderate activity.

BMI: body mass index, eGFR: estimated glomerular filtrate rate, HbA1c: glycated hemoglobin

The prevalence of CVD and ASCVD was numerically higher in secondary (25.6% versus 18.6%) versus primary care (13.6% versus 13.1%); however, differences between the 2 care settings were not compared statistically (Figure 1).

**Figure 2:**
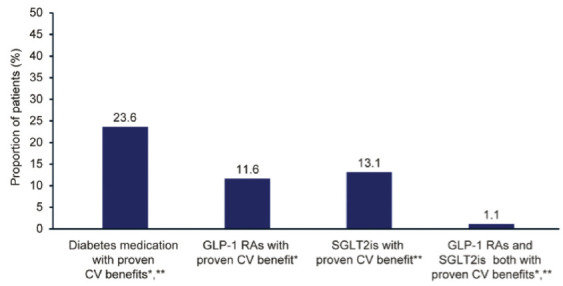
- Glucose-lowering agents with established cardiovascular benefit: use in Saudi Arabia. ^*^Liraglutide, semaglutide, and dulaglutide. ^**^Empagliflozin, canagliflozin, and dapagliflozin. CV: cardiovascular, GLP-1 RA: glucagon-like peptide-1 receptor agonist, SGLT2i: sodium-glucose co-transporter-2 inhibitor

In total, 23.6% of patients were treated with GLAs known to have a CV benefit, of which 11.6% patients were treated with GLP-1 RAs and 13.1% were treated with SGLT2is that had established CV benefit. Additionally, 1.1% of the study population were treated with both a GLP-1 RA and an SGLT2i with established CV benefit (Figure 2).

## Discussion

The estimated CVD prevalence in patients with T2DM in the Saudi Arabian CAPTURE population was 18%, which was markedly lower than the prevalence in the pooled global population (34.8%).^21^ In fact, CVD prevalence was the lowest in Saudi Arabia versus other investigated countries in the CAPTURE study.^21^ In both the Saudi Arabian and pooled global population, the majority of CVD cases were accounted for by ASCVD (84.2% versus 85.8%). Whilst prevalence of CVD was lower in Saudi Arabia than the pooled global population, the use of GLAs that had established CV benefit was found to be consistent with the global population (23.6% versus 21.9%).^21^

Considering the high prevalence of risk factors for CVD in Saudi Arabia,^7^ the estimated prevalence of CVD was unexpectedly low. Data on the prevalence of CVD in the Saudi Arabian population are currently limited; however, one study reported a CVD prevalence between 5601-6600 cases per 100,000 in the general population of Saudi Arabia.^10^ Given that CVD prevalence is usually higher in patients with T2DM compared with the general population,^24^ the CVD prevalence in patients with T2DM in Saudi Arabia would be expected to be higher than the 18% reported here. The low CVD prevalence found in this study may be partly explained by some patient characteristic differences in the Saudi Arabian population versus the pooled global population; patients from Saudi Arabia tended to be younger (median: 58 versus 64 years) and fewer patients had hypertension (55.5% versus 70.1%) compared with the global population.^21^ As well as this, fewer patients from the Saudi Arabian population than in the global population had an eGFR of <59 mL/min/1.73 m^2^ (8.1% versus 21%), and a higher proportion were ‘never smokers’ (80.9% versus 59.5%); all of these characteristics place a patient at a lower risk of developing CVD.^21,25-27^ Another possible explanation for the low CVD prevalence could be patients’ diagnoses being omitted from their medical records at site level due to a lack of coordination between care settings. This is especially plausible as patients in Saudi Arabia can visit other secondary clinics at the first instance, without needing a referral. Lastly, there is a possibility that the Saudi Arabian population is predisposed to protective genes, which could have partly contributed to the low CVD prevalence observed in this study, but a future study is needed to evaluate this possibility.

The prevalence of CVD subtypes was numerically lower in the Saudi Arabian population versus the global sample; for example, coronary heart disease (13.4% versus 17.7%), cerebrovascular disease (1.7% versus 7.2%), and heart failure (0.9% versus 2.4%).^21^ However, similarly, coronary heart disease (comprising the diagnoses of angina, myocardial infarction, other ischemic heart disease, and past revascularization procedures) was the most prevalent CVD subtype in both Saudi Arabia and the global population.^21^ In a study carried out in 2004, the prevalence of coronary artery disease, which comprised the diagnoses of only angina and myocardial infarction, was 5.5% in Saudi Arabia.^28^ In the present study, if only angina and myocardial infarction were evaluated and the other diagnoses of coronary heart disease (other ischemic heart disease and past revascularization procedure) were omitted, the prevalence would be consistent with that found approximately 15 years ago. This could suggest that the prevalence of coronary artery disease has not increased as dramatically as one would anticipate in a country that has experienced such rapid industrialization. However, it is important to bear in mind that the patient population of CAPTURE only involved patients with T2DM, whereas the study carried out in 2004 presented community-based data, therefore comparisons between studies should be drawn cautiously.^28^

Analysis by care setting showed that the CVD prevalence within secondary care was higher than in primary care (25.6% versus 13.6%). The large difference in CVD prevalence in secondary versus primary care may indicate that CVD is underdiagnosed in primary care settings. It may also be indicative of the functionality of the Saudi Arabian healthcare system, whereby patients are not attached to a primary care physician, and as such, most of the patients that develop CVD will not return to their primary care physician after moving to secondary care. A review of the referral of patients with T2DM from primary to secondary and tertiary care in Saudi Arabia revealed that the referral system is poor and there is a lack of clear guidance on when and how to refer patients from one care setting to another.^29^ The inadequate referral of patients with T2DM from primary to secondary/tertiary care could suggest that there may be an underdiagnosis of CVD in the primary care setting. An additional factor that could explain the large difference in CVD prevalence between primary and secondary care could be how the healthcare system is structured in Saudi Arabia, in which patients can choose to see a specialist without first being referred from primary care. Therefore, it is possible that patients with more severe complications, such as CVD, may choose to see a specialist directly, leading to a higher CVD prevalence in secondary care. Additionally, a previous study has found that almost one-third of patients who were treated outside of primary care settings did not have their diagnosis documented at their primary care center due to lack of coordination between care settings, further alluding to the fact that CVD prevalence in primary care may be underrepresented.^30^

Possibly linked to the discrepancies in CVD prevalence between care settings, there were also marked differences in patient characteristics. For example, HbA1c was slightly higher in secondary care (8.4% [68 mmol/mol]) versus primary care (7.8% [62 mmol/mol]). Increased HbA1c is an established risk factor for CVD,^31^ and thus may have contributed to the higher prevalence of CVD in the secondary care setting. Other known risk factors for CVD were also increased in secondary care in comparison with primary, including the proportion of patients with microalbuminuria (33.0% versus 19.6%), the median BMI (30.2 versus 27.9), and the number of patients who had been or currently were smokers (25.6% versus 15.5%).^25-27^ Surprisingly, however, other risk factors were lower in secondary versus primary care, such as hypertension (32.9% versus 68.2%), diabetes duration (8.3 years versus 11.7 years), and a low eGFR (<59 mL/min/1.73 m^2^; 4.4% versus 10.5%).^25^ Therefore, disparities in patient characteristics are unlikely to be the sole factor explaining the difference in CVD prevalence between the 2 care settings.

In addition to, and perhaps a consequence of, the low prevalence of CVD in Saudi Arabia, the prevalence of microvascular complications (neuropathy, retinopathy, and nephropathy) was also unexpectedly low. For example, in the Saudi Arabian population, 13% of patients had neuropathy. This is in contrast with other studies that have reported a prevalence of diabetic neuropathy between 9.6-69.2% in patients with T2DM.^32,33^

### Study limitations

Some patients were required to self-report these complications, which may have led to underreporting.

An unexpected finding was that the prevalence of microvascular complications was higher in primary care than in secondary care settings, whilst CVD prevalence was higher in secondary care. This discrepancy could again be related to lack of coordination between care settings, whereby recorded diagnoses could have been omitted from the patient’s file. Typically, patients with retinopathy are more likely to visit an ophthalmologist than a diabetologist, therefore the prevalence of retinopathy in secondary care may have been underrepresented if the documented diagnosis was not shared with the patient’s secondary care center.

Regarding the usage of GLP-1 RAs and SGLT2is with proven CV benefit, 23.6% of patients in Saudi Arabia were prescribed these medications. This was in line with the global CAPTURE population (21.9%).^21^ It is evident that in both Saudi Arabia and the other countries included in the CAPTURE study, a greater proportion of patients should be treated with these medications to reduce the risk of CV events.^13,20^ Future studies should aim to investigate the trajectory of the uptake of these GLAs with proven CV benefit in Saudi Arabia as the international and local treatment guidelines become more commonplace.

In addition to the limitations already mentioned, other limitations of this study include the lack of statistical analyses between the different groups as well as possible ascertainment bias. Ascertainment bias is likely to have occurred because patients with T2DM and concurrent CVD complications may be more likely to consult their healthcare provider than patients in the general T2DM population. Also, the data herein are limited to the defined study period (December 2018 to September 2019), thus it is likely that CVD prevalence has changed in the intervening time following completion of the study. Finally, data were only collected from 7 sites located in the most developed cities in Saudi Arabia, meaning that the data in this study may not be representative of the whole Saudi Arabian population.

In conclusion, in the CAPTURE study, the prevalence of CVD in patients with T2DM in Saudi Arabia was 18%, which was lower than the pooled global prevalence (35%). The prevalence was unexpectedly low considering the high CVD risk factor prevalence in Saudi Arabia. The low CVD prevalence may be explained by factors such as disparities in patient characteristics, potential genetic predisposition, and poor liaison between different physicians/care settings, leading to potentially inadequate documentation.

## Figures and Tables

**Figure:**
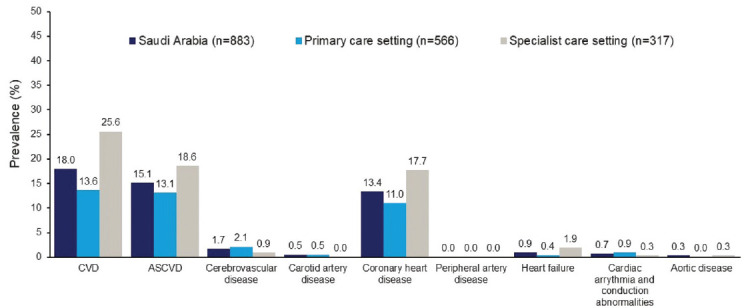
Differences between care settings were not compared statistically. ASCVD: atherosclerotic cardiovascular disease, CVD: cardiovascular disease

## Appendix 1 - List of independent Ethics Committees/Institutional Review Boards.

**Table:**

Sites	IEC/IRB names
Alhada Armed Forces Hospital	Research & Ethics Committee of Armed Forces Hospital, Western Region
King Saud University Medical City, King Saud University	King Saud University Medical City IRB
Specialized Medical Centre Hospital	Specialized Medical Center Hospital IRB
King Abdulaziz University Hospital	King Abdulaziz University Hospital IRB
King Abdulaziz Hospital for National Guard	King Abdullah International Medical Research Center
Diabetes and Endocrinology Center-Buraidah	King Fahd Medical City IRB
Dallah Hospital	King Saud University Medical City IRB

IEC: Independent Ethics Committee, IRB: Institutional Review Board

## Appendix 2 - Definition of atherosclerotic cardiovascular disease and cardiovascular disease subtypes in the CAPTURE study.

**Table:**

Cardiovascular disease		
Atherosclerotic cardiovascular diseases		
Subtypes	Diagnoses used	Further optional details
Cerebrovascular disease*	Ischaemic stroke	N/A
	Haemorrhagic stroke	
	Stroke, unspecified	
	Transient ischaemic attack	
Carotid artery disease*	N/A	N/A
Coronary heart disease*	Myocardial infarction	N/A
	Stable coronary artery disease	Also known as angina pectoris
	Other ischaemic heart disease	N/A
	Past revascularisation procedure	N/A
Peripheral artery disease*	Asymptomatic peripheral artery disease	Low ankle-branchial index (<0.90) or pulse abolition
	Claudication	N/A
	Limb ischaemia	N/A
	Non-traumatic amputation	N/A
Heart failure	Symptomatic	NYHA group II-IV or unknown, LVEF ≥50%, LVEF 40-49%, LVEF <40% or LVEF unknown
	Asymptomatic	NYHA group I, LVEF ≥50%, LVEF 40-49%, LVEF <40%, or LVEF unknown
	Hospitalisation due to heart failure	N/A
Cardiac arrhythmia	Atrial fibrillation, atrial flutter	N/A
	Supraventricular tachycardia	
	Ventricular tachycardia	
	Ventricular fibrillation	
	Bradyarrhythmia, sinus node dysfunction	
	Bradyarrhythmia, atrioventricular block	
Aortic disease	Aortic dissection	N/A
	Aortic aneurysm	
	Thromboembolic aortic disease	

^*^ Cardiovascular disease was categorized as atherosclerotic cardiovascular disease, when a patient had one of these four diagnoses. LVEF: left ventricular ejection fraction, N/A: non-applicable, NYHA: New York Heart Association functional classification.

From original Table S1 from Mosenzon et al. Cardiovasc Diabetol 2021; doi: 10.1186/s12933-021-01344-0, under the Creative Commons Attribution License 4.0; Copyright © 2021, The Author(s).”
